# Antibiotic use for inpatient newborn care with suspected infection: EN-BIRTH multi-country validation study

**DOI:** 10.1186/s12884-020-03424-7

**Published:** 2021-03-26

**Authors:** Ahmed Ehsanur Rahman, Aniqa Tasnim Hossain, Sojib Bin Zaman, Nahya Salim, Ashish K.C., Louise T. Day, Shafiqul Ameen, Harriet Ruysen, Edward Kija, Kimberly Peven, Tazeen Tahsina, Anisuddin Ahmed, Qazi Sadeq-ur Rahman, Jasmin Khan, Stefanie Kong, Harry Campbell, Tedbabe Degefie Hailegebriel, Pavani K. Ram, Shamim A. Qazi, Shams El Arifeen, Joy E. Lawn, EN-BIRTH Study Group, Ahmed Ehsanur Rahman, Tazeen Tahsina, Sojib Bin Zaman, Shafiqul Ameen, Abu Bakkar Siddique, Aniqa Tasnim Hossain, Tapas Mazumder, Jasmin Khan, Taqbir Us Samad Talha, Rajib Haider, Md. Hafizur Rahman, Anisuddin Ahmed, Tanvir Hossain, Qazi Sadeq-ur Rahman, Shams Arifeen, Omkar Basnet, Avinash K. Sunny, Nishant Thakur, Regina Jurung, Anjani Kumar Jha, Bijay Jha, Ram Chandra Bastola, Rajendra Paudel, Asmita Paudel, K. C. Ashish, Nahya Salim, Donat Shamba, Josephine Shabani, Kizito Shirima, Menna Narcis Tarimo, Godfrey Mbaruku, Honorati Masanja, Louise T. Day, Harriet Ruysen, Kimberly Peven, Vladimir Sergeevich Gordeev, Georgia R. Gore-Langton, Dorothy Boggs, Stefanie Kong, Angela Baschieri, Simon Cousens, Joy E. Lawn

**Affiliations:** 1 Maternal and Child Health Division, International Centre for Diarrhoeal Disease Research, Bangladesh (icddr,b), 68 Shahid Tajuddin Ahmed Sarani, Mohakhali, Dhaka, Bangladesh; 2 Department of Health Systems, Impact Evaluation and Policy, Ifakara Health Institute (IHI), Dar es Salaam, Tanzania; 3 Department of Paediatrics and Child Health, Muhimbili University of Health and Allied Sciences (MUHAS), Dar Es Salaam, Tanzania; 4 International Maternal and Child Health, Department of Women’s and Children’s Health, Uppsala University, Uppsala, Sweden; 5 Maternal, Adolescent, Reproductive & Child Health (MARCH) Centre, London School of Hygiene & Tropical Medicine, London, UK; 6 Florence Nightingale Faculty of Nursing, Midwifery & Palliative Care, King’s College London, London, UK; 7 University of Edinburgh, Edinburgh, UK; 8 UNICEF, New York, USA; 9 USAID (contractor), Washington, DC USA; 10 World Health Organization, Geneva, Switzerland

**Keywords:** Newborn, Neonatal infections, Sepsis, Antibiotics, Coverage, Quality of care, Hospital records, Survey, Validity, Antimicrobial resistance

## Abstract

**Background:**

An estimated 30 million neonates require inpatient care annually, many with life-threatening infections. Appropriate antibiotic management is crucial, yet there is no routine measurement of coverage. The *Every Newborn* Birth Indicators Research Tracking in Hospitals (EN-BIRTH) study aimed to validate maternal and newborn indicators to inform measurement of coverage and quality of care. This paper reports validation of reported antibiotic coverage by exit survey of mothers for hospitalized newborns with clinically-defined infections, including sepsis, meningitis, and pneumonia.

**Methods:**

EN-BIRTH study was conducted in five hospitals in Bangladesh, Nepal, and Tanzania (July 2017–July 2018). Neonates were included based on case definitions to focus on term/near-term, clinically-defined infection syndromes (sepsis, meningitis, and pneumonia), excluding major congenital abnormalities. Clinical management was abstracted from hospital inpatient case notes (verification) which was considered as the gold standard against which to validate accuracy of women’s report. Exit surveys were conducted using questions similar to The Demographic and Health Surveys (DHS) approach for coverage of childhood pneumonia treatment. We compared survey-report to case note verified, pooled across the five sites using random effects meta-analysis.

**Results:**

A total of 1015 inpatient neonates admitted in the five hospitals met inclusion criteria with clinically-defined infection syndromes. According to case note verification, 96.7% received an injectable antibiotic, although only 14.5% of them received the recommended course of at least 7 days. Among women surveyed (*n* = 910), 98.8% (95% CI: 97.8–99.5%) correctly reported their baby was admitted to a neonatal ward. Only 47.1% (30.1–64.5%) reported their baby’s diagnosis in terms of sepsis, meningitis, or pneumonia. Around three-quarters of women reported their baby received an injection whilst in hospital, but 12.3% reported the correct antibiotic name. Only 10.6% of the babies had a blood culture and less than 1% had a lumbar puncture.

**Conclusions:**

Women’s report during exit survey consistently underestimated the denominator (reporting the baby had an infection), and even more so the numerator (reporting known injectable antibiotics). Admission to the neonatal ward was accurately reported and may have potential as a contact point indicator for use in household surveys, similar to institutional births. Strengthening capacity and use of laboratory diagnostics including blood culture are essential to promote appropriate use of antibiotics. To track quality of neonatal infection management, we recommend using inpatient records to measure specifics, requiring more research on standardised inpatient records.

**Supplementary Information:**

The online version contains supplementary material available at 10.1186/s12884-020-03424-7.

## Key findings

**Table:** 

**What is known and what is new about this study?**
• Neonatal infections, including sepsis, pneumonia and meningitis account for over half a million neonatal deaths annually, yet most of these deaths are avoidable with appropriate antibiotic and supportive care management. Currently, there are no data from surveys or routine health information systems to track coverage of antibiotic treatment for newborn infections. Such data are increasingly important given rising antimicrobial resistance (AMR).
• The *Every Newborn*-Birth Indicators Research Tracking in Hospitals (EN-BIRTH) study aimed to validate selected maternal and newborn indicators, including use of injectable antibiotics for treating inpatient newborns with clinically-defined infections. This is the first study to assess validity of this indicator in exit survey of women’s report, compared to inpatient case notes, and involved more than 1000 neonates in five hospitals in Bangladesh, Nepal and Tanzania.
**Survey – what did we find and what does it mean?**
• *Denominator:* Maternal report of admission of a newborn to the inpatient ward had high sensitivity, but diagnoses of infection or specific infection syndromes were poorly reported, with high rates of “Don’t know” replies.
• *Numerator:* Women’s report consistently underestimated the coverage of injectable antibiotics for treating newborns compared to the coverage defined by inpatient case note records, and specific antibiotic names were rarely reported correctly.
**Gap analysis for quality of care and measurement**
• Inpatient case note records could be used to measure antibiotic coverage, but limited note keeping detail may impede abstracting specifics of antibiotic use (dose, duration, etc.).
• Antibiotic stewardship is an issue in several of the EN-BIRTH study participating hospitals. Shockingly few inpatients (10.6%) had a blood culture done, and even fewer had a lumbar puncture (0.3%) despite a documented clinically-defined infection diagnosis. Importantly, in Nepal, there was a much higher rate of blood cultures in comparison to the other sites (81.7%). Few neonates received recommended antibiotics for the minimum duration of time. Both these practices are likely to contribute to overtreatment and/or inappropriate use of antibiotics, and may fuel AMR rates.
**What next and research gaps?**
• Exit interview surveys of women’s report are not accurate for measuring coverage of antibiotics for neonatal infections, for denominator and especially for numerator regarding specific antibiotic names. This is consistent with previous research regarding antibiotics for childhood pneumonia, where survey report was inaccurate regarding both numerator and denominator. However, women’s report of admission to a neonatal ward holds promise for use in surveys and requires further research. This indicator could be analogous to other “contact” point indicators such as institutional birth, with scope to link with data on quality of care.
• The gap for laboratory investigations of clinically-defined neonatal infections is a major challenge hence wider use of blood cultures and laboratory capacity strengthening are crucial and success in one of the five EN-BIRTH study hospitals shows this is possible in LMICs. Neonatal sepsis diagnostic innovation is an important investment gap especially given increasing AMR.
• Implementation research is required to assess feasibility and utility of a ward register for inpatient small and sick newborn care focusing on major neonatal conditions including infection diagnoses and antimicrobial use, as well as the transition into electronic systems, with a minimal core dataset.

## Background

Infections, including sepsis, pneumonia and meningitis, account for one-third of all newborn deaths globally [1, 2]. More than half a million newborns die every year due to infections, and the majority of these deaths occur in low- and middle-income countries (LMICs), mainly in south Asia and sub-Saharan Africa [3–5]. Without significantly accelerating the annual rate of reduction, global efforts will not be enough to achieve the ambitious Sustainable Development Goal (SDG) target of reducing the neonatal mortality rate to ≤ 12 per 1000 live births by 2030 [6–8]. Mortality is only the tip of this iceberg of disease burden, as there are an estimated 7 million episodes of possible severe infections among newborns every year, of which around 3.5 million are in south Asia and 2.6 million in sub-Saharan Africa [9]. In total estimated 30 million small and sick newborns require hospital admission, many of whom are given antibiotics [10]. The rate of hospital-acquired infections and antimicrobial resistant (AMR) infections among newborns may further increase due to the trend towards rapid increase in the proportion of births in health facilities in LMICs, and high use of antibiotics often without blood cultures or other diagnostics [11, 12].

Early appropriate management of neonatal infections is critical for newborn survival. The World Health Organization (WHO) recommends inpatient management of infections among newborns with injectable antibiotics [13]. Early administration of appropriate injectable antibiotics with supportive care could avert hundreds of thousands of deaths a year [14–16]. However, substantial gaps exist between such recommendations and implementation [17–19], and there is a dearth of studies to inform measuring the coverage and quality of inpatient management of infections, particularly in LMIC contexts.

Accurate data are crucial to track progress towards the SDGs and the global vision to end all preventable maternal and newborn mortality as well as stillbirths. The *Every Newborn* Action Plan (ENAP) identified a set of core and additional indicators to be measured globally to monitor and track the progress of newborn health. A multi-partner ENAP measurement improvement roadmap was developed to validate these indicators [20]. The proportion of hospitalized neonates with clinically diagnosed infections who received injectable antibiotics [denoted in this manuscript as “coverage” of injectable antibiotics in this target group] was included in the roadmap as one of the core coverage indicators for global monitoring after validation and feasibility testing.

**Fig. 1:**
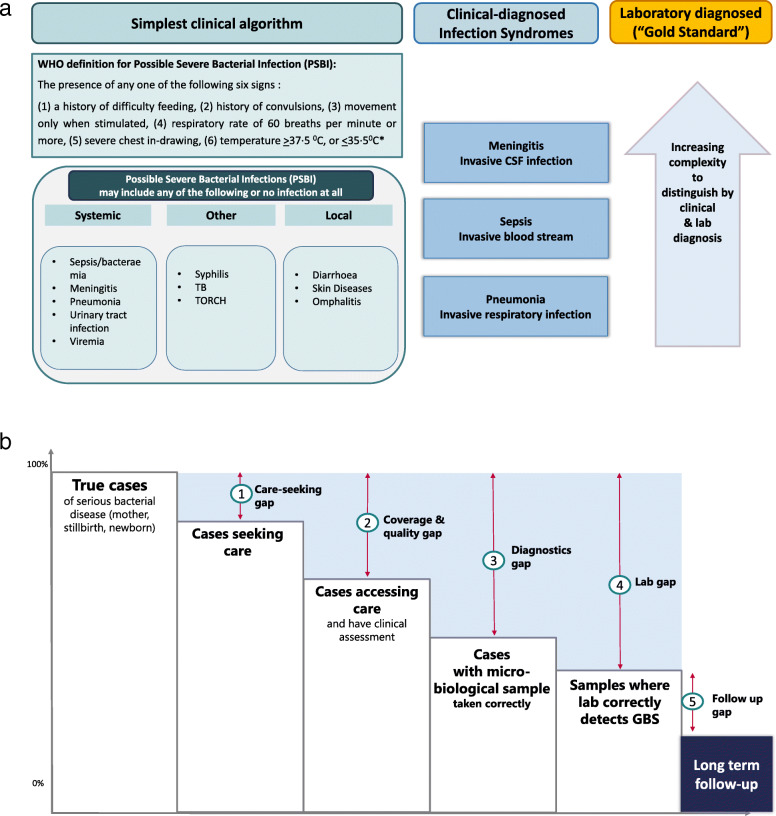
Case definitions and diagnosis for neonatal infections. **a** Approaches to diagnosis of neonatal infection, from simplest clinical algorithm, to infection syndromes through to gold standard with laboratory confirmation (Figure adapted from Seale et al, Lancet Infect Dis, 2014) [9]. **b** Case ascertainment for neonatal invasive bacterial disease showing the cascade affecting gold standard detection (Figure adapted from Lawn et al, Clinical Infectious Diseases, 2017) [23]

The first step towards robust measurement of coverage is applying standardised case definitions. An important challenge is that neonatal infections are primarily defined based on symptoms and signs, which are often poorly codified, and sick neonates commonly have multi-organ dysfunction [21]. For outpatient and primary care settings WHO recommends a simplified clinical algorithm [22], designed to be highly sensitive and non-specific and hence the majority of cases likely have no bacterial infection [4]. For inpatient care of neonatal infections, with more experienced clinicians, a syndromic classification is used to try to separate sepsis, pneumonia and meningitis (Fig. 1a) and this inpatient context is the focus of the *Every Newborn* - Birth Indicators Research Tracking in Hospitals (EN-BIRTH) study. Blood culture remains the gold standard diagnosis, even though this may be negative in more than half of cases where skilled clinicians are confident of the diagnosis (Fig. 1b) [9, 23]. Importantly, meningitis cannot be distinguished from sepsis by clinical examination alone in a neonate and relies on consistent use of lumbar puncture. Laboratory diagnosis require at least a basic microbiological culture capacity, but to get more accurate measures for fastidious organisms such as Group B Streptococcus, requires specific approaches for culturing and more capacity [23].

The next step is that coverage data should be routinely available at scale in either surveys or routine health management information systems (HMIS). Many LMICs still depend on population-based surveys such as The Demographic and Health Surveys (DHS) Program and Multiple Indicator Cluster Surveys (MICS) to report coverage for health care use including for management of childhood illnesses [24, 25]. One important issue is the challenge of measuring denominators of clinical need, especially in surveys. Previous research found challenges with accuracy of recall of denominators regarding childhood infections, notably pneumonia [26]. Another study found that survey-reported pneumonia had low validity with low true positive cases with high levels of false positives [27]. Studies have shown that more extended recall periods (classically 2–5 years in for MICS/DHS) for caregiver-reported symptoms of childhood illnesses especially for newborns, are prone to recall bias and recall error [28, 29].

Despite increasing opportunities to improve measurement in routine facility-based information systems, there has been little research on coverage validity for newborn care. This is an important opportunity, given that ~ 80% of the world’s births are now in facilities [30] coverage for newborn care has also increased, and many LMICs are adopting different digital innovations and transforming paper-based reporting system to digital platforms [31, 32]. However, the majority of the record-keeping system and registers are still paper-based, including for inpatient care. Moreover, collating other relevant data from various care areas make the documentation process more strenuous. In settings with limited resources, inpatient records are mostly based on case notes/case recording forms, that are not standardised and may have variable data quality [33].

As yet, no published studies have assessed the validity of survey report for clinically-defined neonatal infections, to inform the use of surveys to collect coverage data on this important aspect of universal health coverage, or explored feasibility for capture in facility data systems.

## Objectives

This paper is part of a supplement based on the EN-BIRTH multi-country validation study, *‘Informing measurement of coverage and quality of maternal and newborn care’*, and focuses on injectable antibiotic treatment of clinically-defined neonatal infections (sepsis, meningitis and pneumonia) amongst inpatients, addressing the following objectives:

1. **VALIDATION of women’s report through exit survey** to determine the accuracy/validity for
  - ***Denominator options*:** The following denominator options were assessed-
    Option d1- Reported the baby was admitted to newborn ward
    Option d2- Reported the baby was admitted and had any infection
    Option d3- Reported the baby was admitted and had any one of the clinically-defined infection syndromes. i.e. sepsis, pneumonia, meningitis
  - ***Numerator options:*** The following numerator options were assessed-
    Option n1- Reported the baby received any injection/antibiotic
    Option n2- Reported the baby received any injection/antibiotic and reported the antibiotic name
2. **QUALITY GAP ANALYSES for injectable antibiotic use** to assess the gaps in coverage, quality and measurement from case note verification.
3. **BARRIERS AND ENABLERS** to understand the barriers and enablers of documentation practices from qualitative interviews.

## Methods

**Fig. 2:**
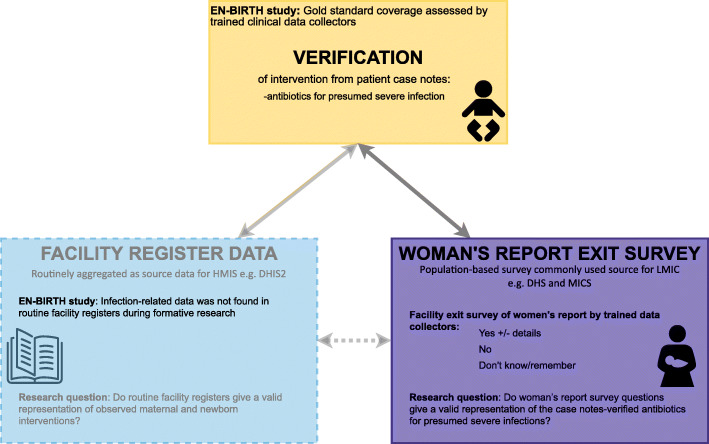
EN-BIRTH study antibiotic coverage validation design comparing case note verification with exit interview survey [34]

The EN-BIRTH study was conducted in five referral hospitals: Maternal and Child Health Training Institute (MCHTI), Azimpur and Kushtia General Hospital in Bangladesh (BD), Pokhara Academy Health Sciences in Nepal (NP), and Muhimbili National Hospital and Temeke District Hospital in Tanzania (TZ) (Additional file 1). These hospitals were selected since all the maternal, and neonatal interventions of interest were available. The participants were consenting women (primary caregivers of newborns) whose baby was admitted to the hospital inpatient department (newborn and paediatric wards) and treated for neonatal infection. Detailed information regarding the research protocol, methods, and analysis were published separately [34, 35]. In this study, we compared clinically-defined neonatal infection verified through abstraction of data from inpatient case notes (gold standard) with women’s report collected through exit surveys (Fig. 2).

### Data collection

We adopted both quantitative and qualitative methods of data collection to address the study objectives. Data collection took place between July 2017 and July 2018. Details regarding the clinical management practices were verified by abstracting data from hospital inpatient case notes/case recording forms with a structured checklist. Exit surveys were conducted with a structured questionnaire to capture women’s report before discharge. These quantitative data collection tools were developed by team members from Bangladesh, Nepal, Tanzania and UK based on the global guidelines and validated tools [13, 36, 37]. The data collection tools were adapted to reflect country settings and contexts (health systems, language, culture, etc.) through formative research. Trained data collectors collected data using a custom-built android tablet-based electronic data capture system specially designed for the EN-BIRTH study. Separate researchers were assigned to verify the hospital inpatient case notes, in addition to those assigned to conduct the exit surveys. Around 5% of the case note verifications were re-checked by field supervisors to monitor the reliability of data collection.

In-depth interviews and focus group discussions were conducted by trained qualitative researcher to explore potential barriers and enablers related to documentation practices. Qualitative data collection tools were informed by the Performance of Routine Information System Management (PRISM) conceptual framework [38, 39]. We obtained ethical approval from the institutional review boards in all operating counties in addition to the London School of Hygiene & Tropical Medicine (Additional file 2).

### Eligibility criteria

All babies aged ≤28 days at admission, weighing > 1500 grammes (g) at admission or discharge, or gestational age > 32 weeks, receiving inpatient management for clinically-defined infections, i.e. sepsis, pneumonia, meningitis were included for analysis in this paper. Babies with an obvious major congenital abnormality, neonatal encephalopathy (“severe asphyxia”) were excluded at recruitment. All inclusion and exclusion criteria were based on the data abstracted from hospital inpatient records.

### Data analyses

Results are reported in accordance with STROBE Statement checklists for observational studies (Additional file 3). We reported the background characteristics of newborns treated for clinically-defined infections and the women (primary caregivers) who were successfully interviewed. Socioeconomic asset scores were generated using the standard principal component analysis procedure. The EN-BIRTH larger dataset was used for the assignment of wealth quintile to the neonatal infection cases [34, 35].

We reported the antibiotic coverage among newborns treated for clinically-defined infections for the following scenarios based on the hospital inpatient case note verification: any injectable antibiotic, any WHO recommended injectable antibiotic, any recommended injectable antibiotic for 2 days, any recommended injectable antibiotic for 7 days. Survey-reported antibiotic coverage was reported for two questions: general- reported any injection or antibiotic was given, and specific- reported the name of a specific antibiotic. We used descriptive statistics to report all point prevalence estimates with 95% confidence intervals. We reported all estimates separately for each of the five facilities, as well as pooled estimates through random effect models with heterogeneity statistics (I^2^ and τ^2^).

We conducted individual level validation analyses of women’s report for the different denominator and numerator options. Hospital inpatient case note verification was considered as gold standard, and women’s report during the exit survey was regarded as the ‘Test’ during this analysis (Fig. 2). The denominator options included whether the women could correctly report if the baby was admitted in the hospital (option d1), if the baby was admitted and had any infection (option d2), and if the baby was admitted and had any clinically-defined infection (option d3). The numerator options included whether the women could correctly report if their baby received an injection or antibiotics (option n1) and whether the women could specifically report the name of an antibiotic (option n2).

For validity measures, sensitivity and specificity were reported with 95% confidence interval for each of the selected hospital separately. Exit survey reported “Don’t know” category was considered as “No” during this analysis. Also, we reported the percent-agreement between the case note verification and the exit survey. Sensitivity and specificity analyses were only performed if the column total counts in two-way tables exceeded 10. For denominator validation, we did not report the sensitivity, specificity and percent agreement as we only had newborns treated for clinically-defined infections, i.e. no “true negatives.”

Structured Query Language (SQL) server was used to store and manage data. We used Stata (version 14) for conducting all quantitative analysis. NVivo 12 software was used to manage qualitative data during analysis.

## Results

**Fig. 3:**
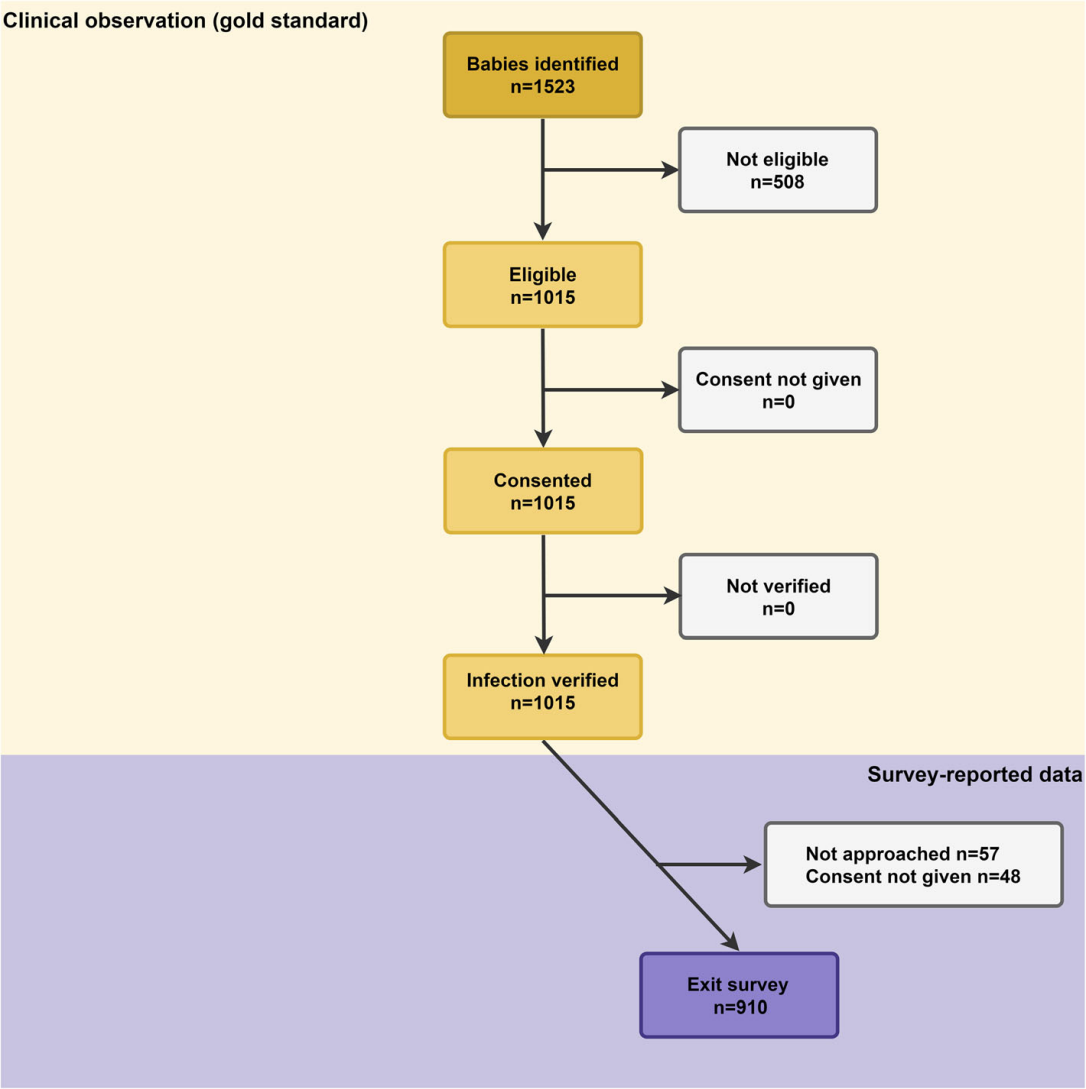
EN-BIRTH study flow diagram for newborns treated with severe infections (*n* = 1015)

A total of 1015 cases were selected based on the inclusion and exclusion criteria (from 1523 recruited), among which 409 newborns were from Bangladesh, 344 were from Nepal, and 262 were from Tanzania. Among 1015 eligible cases, 910 women (primary caregivers of the newborns) were successfully interviewed, 57 women were lost to follow up, and 48 women did not consent to participate in the study. Figure 3 summarises the selection process, regarding the distribution of different inclusion and exclusion criteria among the overall sample.

Background characteristics, clinical history and results of physical examination of the newborns on admission as recorded in the hospital inpatient case notes are shown in Table 1. Among all newborns treated for clinically-defined infections, 78.3% in Azimpur BD, 76.9% in Kushtia BD and 75.5% in Muhimbili TZ, and around 99% in Pokhara NP and Temeke TZ were recorded as sepsis cases. Around 20% of the babies in Azimpur BD and Kushtia BD and less than 1% of the newborns in Pokhara NP and Temeke TZ were recorded as pneumonia cases. The majority of the newborns were less than 7 days of age, except in Muhimbili TZ (24.5%), where the majority were aged between 7 and 13 days (46.9%). 36.3% of the newborns in Kushtia BD and 12.3% in Muhimbili TZ had a history of low birthweight (< 2500 g). Weight on admission was not recorded for less than 10% cases in Azimpur BD and Kushtia BD, 22.4% in Muhimbili TZ and more than 70% in Pokhara NP and Temeke TZ.

**Table 1:** Characteristics of newborns in inpatient wards, case note verification, EN-BIRTH study (*n* = 1015 children)

	Bangladesh		Nepal	Tanzania	
	Azimpur Tertiary	Kushtia District	Pokhara Regional	Temeke Regional	Muhimbili National
	***N*** = 106	***N*** = 303	***N*** = 344	***N*** = 213	***N*** = 49
	n (%)	n (%)	n (%)	n (%)	n (%)
**Age**					
≤ 6 days	67 (63.2)	151 (49.8)	259 (75.3)	153 (71.8)	12 (24.5)
7–13 days	17 (16)	60 (19.8)	42 (12.2)	34 (16)	23 (46.9)
14–20 days	13 (12.3)	42 (13.9)	19 (5.5)	13 (6.1)	9 (18.4)
21–28 days	9 (8.5)	50 (16.5)	24 (7)	13 (6.1)	5 (10.2)
**Sex**					
Male/Boy	59 (55.7)	183 (60.4)	225 (65.4)	127 (59.6)	30 (61.2)
**Birth weight**					
1500–2000 g	7 (6.6)	38 (12.5)	15 (4.4)	14 (6.6)	4 (8.2)
2000–2500 g	20 (18.9)	72 (23.8)	54 (15.7)	30 (14.1)	2 (4.1)
2500+ g	65 (61.3)	165 (54.5)	263 (76.5)	165 (77.5)	40 (81.6)
Others	14 (13.2)	28 (9.2)	12 (3.5)	4 (1.9)	3 (6.1)
**Weight at admission**					
1500–2000 g	5 (4.7)	41 (13.5)	3 (0.9)	3 (1.4)	0 (0)
2000–2500 g	26 (24.5)	81 (26.7)	12 (3.5)	7 (3.3)	5 (10.2)
2500+ g	67 (63.2)	157 (51.8)	80 (23.3)	29 (13.6)	33 (67.3)
Others	8 (7.5)	24 (7.9)	249 (72.4)	174 (81.7)	11 (22.4)
**History**					
Not Feeding Well	43 (40.6)	37 (12.2)	42 (12.2)	77 (36.2)	18 (36.7)
Lethargy/reduced consciousness	6 (5.7)	2 (0.7)	16 (4.7)	14 (6.6)	9 (18.4)
Convulsion	3 (2.8)	8 (2.6)	12 (3.5)	21 (9.9)	7 (14.3)
Fever	44 (41.5)	25 (8.3)	211 (61.3)	127 (59.6)	20 (40.8)
Respiratory distress or fast breathing	36 (34)	35 (11.6)	45 (13.1)	20 (9.4)	7 (14.3)
**Physical examination**					
Fever (> 38 degree)	28 (26.4)	298 (98.3)	172 (50)	81 (38)	10 (20.4)
Hypothermia (< 35 degree)	3 (2.8)	0 (0)	0 (0)	1 (0.5)	0 (0)
Respiratory Rate (> 60/min)	40 (37.7)	23 (7.6)	135 (39.2)	29 (13.6)	9 (18.4)
Bulging Fontanels	0 (0)	0 (0)	0 (0)	2 (0.9)	2 (4.1)
Umbilical redness and draining pus	8 (7.5)	0 (0)	3 (0.9)	5 (2.3)	3 (6.1)
Skin Pustules	2 (1.9)	2 (0.7)	11 (3.2)	3 (1.4)	3 (6.1)
**Diagnosis at admission**					
Sepsis	83 (78.3)	233 (76.9)	341 (99.1)	211 (99.1)	37 (75.5)
Pneumonia	23 (21.7)	70 (23.1)	1 (0.3)	1 (0.5)	8 (16.3)
Meningitis	0 (0)	0 (0)	2 (0.6)	1 (0.5)	4 (8.2)
**Baby’s condition at discharge**					
Alive	106 (100)	272 (89.8)	342 (99.4)	196 (92)	45 (91.8)
Death	0 (0)	1 (0.3)	1 (0.3)	5 (2.3)	4 (8.2)
Not Recorded	0 (0)	30 (9.9)	1 (0.3)	12 (5.6)	0 (0)

Additional file 4 presents the characteristics of the mothers of the newborns who participated in the exit survey—the majority of these mothers were aged between 20 and 29 years. 28.8% women completed secondary education in Kushtia BD and 61.0% in Pokhara NP.

### Objective 1: Denominator and numerator validation

Table 2 presents the denominator validation results of women’s reports during the exit survey, which were compared with hospital inpatient case note verification. Among the 910 women surveyed, 98.8% could report their baby was admitted in the hospital, which was consistent across all facilities. 47.1% of women could report their baby was admitted in the hospital and had any infection, which varied across different hospitals, ranging from 17.1% (6.5–33.6%) in Muhimbili TZ to 75.4% (70.1–80.1%) in Kushtia BD. Only 30.4% (10.0–55.91%) of women could report if their baby was admitted in the hospital and had a clinically-defined infection, which also varied substantially across different hospitals, ranging from 11.4% (3.2–26.7%) in Muhimbili TZ to 70.4% (64.9–75.5%) in Kushtia BD.

**Table 2:** Denominator validation results for coverage of injectable antibiotics, women’s exit interview survey, EN-BIRTH study (*n* = 901)

	Country	Hospital	Survey Reported Coverage		Don’t Know Response
			N	% (95% CI)	%
**Baby admitted in hospital**	Bangladesh	Azimpur Tertiary	103	99 (93, 99.8)	0
		Kushtia District	301	99 (96.9, 99.0)	0
	Nepal	Pokhara Regional	316	97.2 (94.6, 98.5)	0.32
	Tanzania	Temeke Regional	146	99.3 (95.2, 99.9)	0.68
		Muhimbili National	35	100	0
	**All sites pooled**	**Random effects estimate**	901	98.8 (97.8, 99.5)	0.2
**Baby admitted in hospital and had any infection**	Bangladesh	Azimpur Tertiary	103	41.7 (32.1, 51.8)	0
		Kushtia District	301	75.4 (70.1, 80.1)	7.64
	Nepal	Pokhara Regional	316	58.8 (53.2, 64.3)	2.53
	Tanzania	Temeke Regional	146	38.3 (30.4, 46.7)	4.11
		Muhimbili National	35	17.1 (6.5, 33.6)	8.57
	**All sites pooled**	**Random effects estimate**	901	47.1 (30.1, 64.5)	3.45
**Baby admitted in hospital and had a presumed severe infection**	Bangladesh	Azimpur Tertiary	103	30.1 (21.5, 39.9)	2.91
		Kushtia District	301	70.4 (64.9, 75.5)	11.3
	Nepal	Pokhara Regional	316	17.4 (13.3, 22.0)	6.33
	Tanzania	Temeke Regional	146	26.7 (19.7, 34.6)	4.79
		Muhimbili National	35	11.4 (3.2, 26.7)	8.57
	**All sites pooled**	**Random effects estimate**	901	30.4 (10.0, 55.9)	6.48

**Fig. 4:**
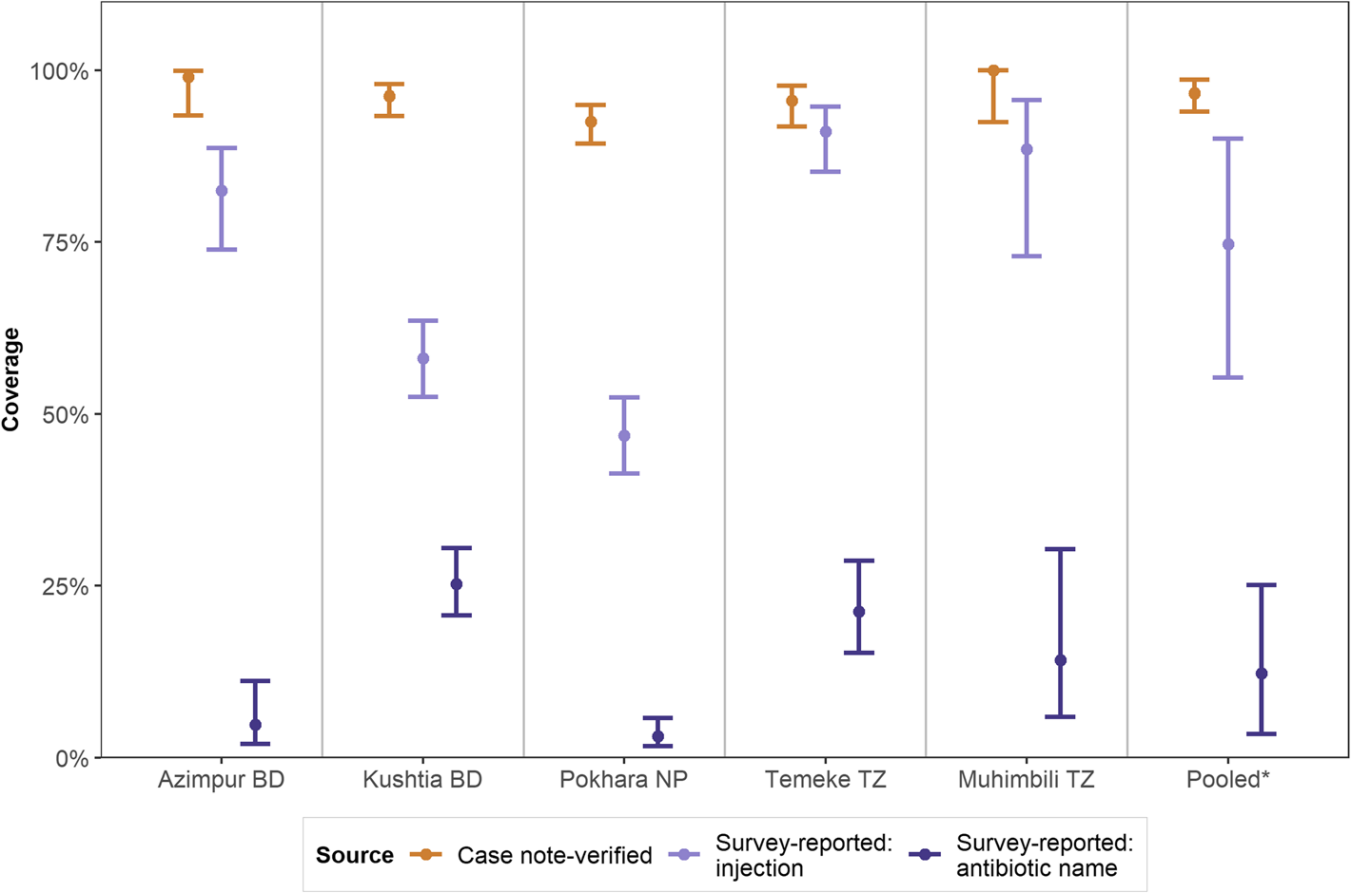
Coverage of antibiotics − newborn care inpatient wards, EN-BIRTH study. *Random effects

Overall, 74.7% (55.3–90.1%) of women could report their baby received any antibiotics/injections during their hospital stay: more than 80% in Azimpur BD, Temeke TZ and Muhimbili TZ; whereas only 58.1% in Kushtia BD and 46.8% in Pokhara NP (Fig. 4). Around one-third of women in Kushtia BD and one-fourth of women in Pokhara NP mentioned that they did not know or remember whether their baby received any antibiotic/injection. The sensitivity of women’s report whether their babies received any antibiotic/injection was 75.9% (Table 3, Additional files 5 and 6).

**Table 3:** Individual-level numerator validation in exit survey report of injectable antibiotics coverage, EN-BIRTH study (*n* = 901)

	Bangladesh				Nepal		Tanzania				All sites pooled (Random Effects) % and 95 CI	
	Azimpur Tertiary		Kushtia District		Pokhara Regional		Temeke Regional		Muhimbili National			
**5.1 Neonatal Infection - Antibiotic/Injection - Survey reported**												
Observer coverage %	99.0	(93.4, 99.9)	96.3	(93.4, 97.9)	92.6	(89.3, 95.0)	95.6	(91.8, 97.7)	100.0	(92.5, 100.0)	96.7	(94.0, 98.6)
Survey reported coverage %	82.5	(73.9, 88.7)	58.1	(52.5, 63.6)	46.8	(41.4, 52.4)	91.1	(85.2, 94.8)	88.6	(72.9, 95.7)	74.7	(55.3, 90.1)
"Don’t know” responses %	9.7	(5.3, 17.2)	35.2	(30.0, 40.8)	25.0	(20.5, 30.1)	6.8	(3.7, 12.3)	11.4	(4.3, 27.1)	16.9	(7.4, 29.2)
Sensitivity % (95% CI)	†	†	57.8	(51.8, 63.6)	47.8	(41.9, 53.7)	†	†	†	†	75.9	(55.6, 91.6)
Specificity % (95% CI)	‡	‡	54.5	(23.4, 83.3)	62.5	(40.6, 81.2)	‡	‡	‡	‡	‡	‡
Percent agreement (TP+TN)/n %	84.2	(75.6, 90.7)	57.7	(51.8, 63.4)	48.9	(43.2, 54.6)	90.8	(84.9, 95.0)	88.6	(73.3, 96.8)	75.3	(56.4, 90.2)
**5.2 Neonatal Infection - Antibiotic name - Survey reported**												
Observer coverage %	99.0	(93.4, 99.9)	96.3	(93.4, 97.9)	92.6	(89.3, 95.0)	95.6	(91.8, 97.7)	100.0	(92.5, 100.0)	96.7	(94.0, 98.6)
Survey reported coverage %	4.9	(2.0, 11.2)	25.2	(20.6, 30.5)	3.2	(1.7, 5.8)	21.2	(15.3, 28.7)	14.3	(6.0, 30.4)	12.3	(3.5, 25.1)
"Don’t know” responses %	9.7	(5.3, 17.2)	35.2	(30.0, 40.8)	25.0	(20.5, 30.1)	6.8	(3.7, 12.3)	11.4	(4.3, 27.1)	16.9	(7.4, 29.2)
Sensitivity % (95% CI)	†	†	26.2	(21.2, 31.8)	3.5	(1.7, 6.3)	†	†	†	†	12.7	(3.7, 25.6)
Specificity % (95% CI)	‡	‡	90.9	(58.7, 99.8)	100.0	(85.8, 100.0)	‡	‡	‡	‡	‡	‡
Percent agreement (TP+TN)/n %	5.9	(2.2, 12.5)	28.7	(23.6, 34.2)	10.9	(7.6, 14.8)	23.9	(17.2, 31.8)	14.3	(4.8, 30.3)	16.1	(8.0, 26.2)

^†‡^ Validity statistics suppressed where < 10 count in either column of two-by-two table

12.3% (3.5–25.1%) of women could report the specific name of an antibiotic. 35.2% of women in Kushtia BD and 25.0% of women in Pokhara NP mentioned that they did not know or remember the specific name of the antibiotic. The sensitivity of reporting the name of the specific antibiotic was only 12.7%.

### Objective 2: Gaps in coverage, quality and measurements

Table 4 describes the diagnostic practices received by newborns treated for infection, according to the hospital inpatient case note verification. Documentation of blood culture being performed was available only among 10.6% of newborns who were treated for clinically-defined infections. The rate was less than 5% in Bangladesh and Tanzania and 81.7% in Nepal. Less than 1% of newborns had any documented evidence of a lumbar puncture being performed. Among the seven cases which had on admission diagnosis of meningitis, a lumbar puncture was performed in only three cases (data not shown). Only one-fifth of all newborns treated for infection had any documented evidence of high white blood cell (WBC) count.

**Table 4:** Laboratory investigations and diagnostics, case note verification, EN-BIRTH study (*n* = 1015)

	Bangladesh		Nepal	Tanzania		All sites pooled (Random Effects)
	Azimpur Tertiary	Kushtia District	Pokhara Regional	Temeke Regional	Muhimbili National	
	***N*** = 106	***N*** = 303	***N*** = 344	***N*** = 213	***N*** = 49	***N*** = 1015
	n (%)	n (%)	n (%)	n (%)	n (%)	%
**Confirmatory Lab Diagnosis**						
Blood Culture Done	2 (1.9)	2 (0.7)	281 (81.7)	1 (0.5)	2 (4.1)	10.6
Blood Culture Positive	0 (0)	0 (0)	206 (59.9)	0 (0)	1 (2)	5
LP Done	0 (0)	0 (0)	5 (1.5)	0 (0)	2 (4.1)	0.3
LP CSF Appearance Positive	0 (0)	0 (0)	1 (0.3)	0 (0)	0 (0)	0
LP CSF Culture Positive	0 (0)	0 (0)	1 (0.3)	0 (0)	0 (0)	0
LP CSF Clinical Appearance Positive or Culture Positive	0 (0)	0 (0)	2 (0.58)	0 (0)	1 (2.0)	0.1
Either Blood Culture Positive OR CSF Positive	0 (0)	0 (0)	207 (60.2)	0 (0)	2 (4.1)	5.5
**Other Supportive Lab Diagnosis**						
CBC Done	5 (4.7)	9 (3)	309 (89.8)	1 (0.5)	32 (65.3)	25.7
WBC Count High	1 (0.9)	0 (0)	192 (55.8)	1 (0.5)	15 (30.6)	10.3
Either Blood Culture Positive or CSF Positive or WBC high	1 (0.9)	0 (0)	277 (80.5)	1 (0.5)	16 (32.7)	14.2

*LP* lumbar puncture, *CSF* Cerebrospinal fluid, *CBC* Complete blood count, *WBC* White blood cell

Table 5 presents the use of different types of antibiotic in various hospitals according to the case note verification. The choice of antibiotic differed across different hospitals despite the high coverage of antibiotics across all sites. In all hospitals except Kushtia BD, ampicillin (63.4–90.6% across facilities) and gentamicin (69.4–92.5% across facilities) were the most commonly used antibiotics. In Kushtia BD, gentamicin (71.6% with CI 66.1–76.6%), ceftazidime (69.3% with CI 63.7–74.4%) and meropenem (28.1% with CI 23.1–33.5%) were the most frequently used. In addition to ampicillin and gentamicin, ampicillin-cloxacillin (29.6% with CI 23.5–36.1%) was one of the most commonly used in antibiotics in Temeke TZ.

**Table 5:** Injectable antibiotic use and coverage, case note verification, EN-BIRTH study (*n* = 1015)

Name of antibiotic	Bangladesh		Nepal	Tanzania	
	Azimpur Tertiary	Kushtia District	Pokhara Regional	Temeke Regional	Muhimbili National
	***N*** = 106	***N*** = 303	***N*** = 344	***N*** = 213	***N*** = 49
	n (% - CI)	n (% - CI)	n (% - CI)	n (% - CI)	n (% - CI)
Amikacin	4 (3.8 – (1.0–9.4))	44 (14.5– (10.7–19.0))	55 (16 – (12.2–20.3))	0 (0)	0 (0)
Ampicillin	96 (90.6 – (83.3–95.3))	0 (0)	257 (74.7 – (69.7–79.2))	135 (63.4 – (56.5–69.8))	33 (67.3– (52.4–80.0))
Ampicillin-Cloxacillin	0 (0)	0 (0)	0 (0)	63 (29.6 – (23.5–36.1))	0 (0)
Amoxycillin-Cloxacillin	0 (0)	0 (0)	0 (0)	0 (0)	1 (2 – (0.0–10.8))
Azithromycin	0 (0)	1 (0.3 – (0.0–1.8))	0 (0)	0 (0)	0 (0)
Aztreonam	0 (0)	0 (0)	1 (0.3 – (0.0–1.6))	0 (0)	0 (0)
Azoxystrobin (fungicide)	0 (0)	4 (1.3 – (0.3–3.3))	0 (0)	0 (0)	0 (0)
Cefaclor	0 (0)	1 (0.3 – (0.0–1.8))	0 (0)	0 (0)	0 (0)
Cefdinir-Flucloxacillin	0 (0)	0 (0)	1 (0.3 – (0.0–1.6))	0 (0)	0 (0)
Cefixime	0 (0)	0 (0)	23 (6.7 – (4.2–9.8))	0 (0)	0 (0)
Cephalexin	0 (0)	1 (0.3 – (0.0–1.8))	0 (0)	0 (0)	0 (0)
Cefotaxime	0 (0)	2 (0.7 – (0.0–2.3))	38 (11 – (7.9–14.8))	1 (0.5 – (0.0–2.5))	1 (2 – (0.0–10.8))
Ciprofloxacin	0 (0)	0 (0)	0 (0)	0 (0)	4 (8.2 – (2.2–19.6))
Cloxacillin	2 (1.9 – (0.2–6.6)	0 (0)	0 (0)	0 (0)	0 (0)
Cefepime	2 (1.9 - (0.2–6.6))	1 (0.3 – (0.0–1.8))	1 (0.3 – (0.0–1.6))	0 (0)	0 (0)
Ceftriaxone	1 (0.9 – (0.02–5.1))	19 (6.3 – (3.8–9.6))	0 (0)	9 (4.2 – (1.9–7.8)	22 (44.9 – (30.6–59.7))
Flucloxacillin	1 (0.9 – (0.02–5.1))	11 (3.6 – (1.8–6.4))	14 (4.1 – (2.2–6.7))	0 (0)	0 (0)
Gentamicin	98 (92.5 – (85.7–96.7)	217 (71.6 – (66.1–76.6))	270 (78.5 – (73.7–82.7))	197 (92.5 – (88.0–95.6)	34 (69.4 – (54.5–81.7))
Mexidin	0 (0)	1 (0.3 – (0.0–1.8))	0 (0)	0 (0)	0 (0)
Metronidazole	2 (1.9 – (0.2–6.6))	56 (18.5 – (14.2–23.3))	5 (1.5 – (0.5–3.3))	2 (0.9 – (0.1–3.3))	5 (10.2 – (3.3–22.2))
Moxifloxacin	0 (0)	1 (0.3 – (0.0–1.8))	0 (0)	0 (0)	0 (0)
Meropenem	0 (0)	85 (28.1 – (23.1–33.5))	2 (0.6 - (0.0–2.0))	0 (0)	2 (4.1 (0.5–13.9)
Ofloxacin	0 (0)	0 (0)	1 (0.3 – (0.0–1.6))	0 (0)	0 (0)
Ceftazidime	5 (4.7 – (1.5–10.7))	210 (69.3 – (63.7–74.4))	4 (1.2 – (0.3–2.9)	0 (0)	0 (0)
Tobramycin	0 (0)	0 (0)	2 (0.6 - (0.0–2.0))	0 (0)	0 (0)
Vancomycin	0 (0)	0 (0)	5 (1.5 – (0.5–3.3))	0 (0)	0 (0)
Antibiotic - Unspecified	0 (0)	8 (2.6 – (1.1–5.1))	0 (0)	0 (0)	0 (0)

**Fig. 5:**
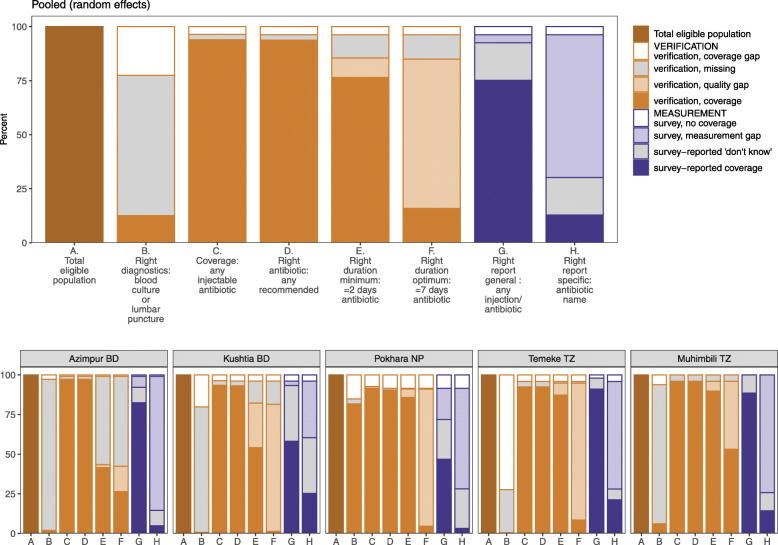
Gaps in coverage, quality and measurements in antibiotic use, EN-BIRTH study. (*n* = 1015 for case note verification and *n* = 910 exit interview survey). *All sites pooled using random effects model

Figure 5 shows the gap analysis for coverage of antibiotic use among newborns treated for clinically-defined infections through the hospital inpatient case note verification (first six stacked bars from left) and gaps in measurements through women’s report at exit survey (the last two stacked bars). Among all the newborns treated for infection, 96.7% had documented evidence of receiving any WHO recommended injectable antibiotic for any duration, 73.3% receiving any recommended injectable for at least 2 days and 14.5% receiving any recommended injectable for at least 7 days.

### Objective 3: Barriers and enablers to documentation practices in hospital inpatient case notes

We identified the following key themes regarding documentation practices in hospital inpatient case notes:

- *Enabler – awareness regarding the importance case note records***:** The health workers, i.e. doctors and nurses responsible for inpatient management of sick newborns, were aware of the importance of documentation in the inpatient case notes and medical record keeping. They acknowledged its importance for reviewing the patient’s condition and taking clinical decisions, communicating and coordinating within the clinical team (doctors’ instructions to the junior doctors and nurses, nurses’ action in response to the guidelines, etc.), and preparing discharge certificates.
- *Enabler – source of information for service reports:* The health services providers, especially the nurses, regularly used case notes as a source of information for preparing different services reports (daily/monthly reports) and disease-specific registries.
- *Barrier – case note design and lack of standardization:* The case notes had a basic structure outlining some key components (particulars of the patient, history, clinical features, laboratory investigations, drugs given, etc.). The design of the case notes varied substantially across countries, and it did not prioritize any standardized documentation of key clinical care elements. Consequently, the documentation practice was dependent on the preference and performance of the clinical service providers, leading to unstandardized documentation of details. The majority of the health service providers felt the need for specific training related to documentation of inpatient care.
- *Barrier – lack of coordination and duplication with other registers:* In addition to case notes, nurses had to maintain other administrative registers such as drugs log, logistic requisition, etc. which also include various patient-related information (which are already available in the case notes) leading to duplication of efforts and documentations. One of the nurses from Bangladesh said:

*“There are too many registers to fill up. Information related to neonatal infection is recorded into the admission book, patient case file, and monthly summary sheet. To do so in a proper way, it needs a considerable amount of time.”*

- Health worker, BD

- *Barrier – clinical workload and documentation responsibilities:* In addition to the clinical duties, the doctors and nurses were separately responsible for filling-in different sections of the case notes. The majority of the health workers felt that their clinical workload was overwhelming and affected the quality of case note documentation.

## Discussion

This analysis, as part of EN-BIRTH study, is the first to validate potential coverage indicator measurement for antibiotic treatment of neonatal infections in hospitalised patients. Based on more than 1000 cases in five hospitals in Bangladesh, Nepal and Tanzania, we validated women’s report during exit survey against information abstracted from hospital inpatient case notes [35]. Given our findings of large measurement gaps of women’s report, we do not recommend incorporating this indicator in widely deployed population-based surveys like DHS and MICS [24, 25].

Maternal report of newborn admission in the inpatient ward had high sensitivity, but specific diagnosis or classifications were poorly reported, with high “Don’t knows”. Infections are a subset of the total neonatal admissions, varying by context and especially by level of facility, with reports between 6 and 68% of all neonatal admissions [40–49]. Using a contact indicator option (admission to a neonatal unit) in household surveys may be useful as marker of care for small and sick newborns, in a similar way that “contact” point indicators such as institutional birth or antenatal care coverage are used. We note that only women whose babies had been admitted were surveyed, so more research is required to also ask those whose baby was not admitted. Importantly this “contact” point indicator would also need to be linked to more detailed diagnosis and treatment information, from inpatient datasets for example.

More detailed questions to try to identify denominators of clinical diagnosis were asked in two ways (any infection, or specific infection syndrome), and both of these performed poorly in survey report. Around half of the women could correctly report whether their baby had any infection, and only around one-third could report any specific infection syndromes (sepsis, meningitis or pneumonia). Moreover, there were wide variations among different hospitals regarding the accuracy of women’s report on the second (if baby admitted in hospital and had any infection) (17.1–75.4%) and the last option (if baby admitted in hospital and had a presumed severe infection (11.4–70.4%). These findings are consistent with the previous studies which reported the challenges of identifying clinical symptoms through household surveys [26, 27, 50]. In our EN-BIRTH study, the sensitivity of women’s report was assessed through exit survey. In contrast, standard surveys like DHS and MICS accept a recall period of 14 days for identifying suspected cases suffering from acute respiratory infections. Since recall bias and recall error increase with longer recall periods, the accuracy of women’s report collected through the last two denominator options in household surveys may be further compromised [28, 29].

The numerators assessed involved questions regarding the use of injectable antibiotics. For use of any antibiotic, the sensitivity was 75.9%, with wide variation between the five participating hospitals. In terms of mothers’ knowledge regarding which antibiotic was given, sensitivity was only 12.7%. This was reasonably consistent across all hospitals. During hospital stay, a sick newborn may require different kinds of injectable drugs in addition to antibiotics [13]. Therefore, the general option (any injection) may overestimate the true coverage of injectable antibiotic for treating newborns with infections. Moreover, antibiotics are often prescribed using trade names (given by the manufacturing companies), making it even more difficult for women to report drug names correctly, and also challenging to differentiate an antibiotic from other drugs during analysis. Effective communication has been underemphasised as part of respectful family-centred care in many LMICs [51]. Such communication gaps might contribute to the limited sensitivity of women’s report and high rates of “Don’t know” responses for this option. Focusing only on hospitalised care might have underestimated the coverage of injectable antibiotic [52]. However, the focus on this study was to assess the validity of hospital inpatient record-keeping and its implications on estimating the antibiotic coverage.

Hospital records are another potential data source for tracking injectable antibiotic use, and could be linked to a “contact” point indicator in surveys to assess effective coverage [53]. We found gaps in the design of hospital inpatient case notes and inconsistencies in documentation practices by various health service providers, and between the hospitals. Introduction of clinical registers for inpatient management of sick newborns may help address such gaps [54] and contribute to better quality of care and patient outcomes [55–57]. Implementation research is required to evaluate the use of novel clinical registers. Shifting towards electronic inpatient records and adopting new technologies designed for resource-poor settings could improve the quality of documentation [58]. However, managing an extensive electronic database can be challenging in any context, and requires adequate resourcing [59, 60].

Antibiotic stewardship is an imperative in every country, and neonates are especially vulnerable to antimicrobial-resistant pathogens and more likely to die if infected [61]. There were gaps regarding the use of recommended antibiotics as included hospitals used around 30 types of injectable antibiotics for treating newborns. Furthermore, there are concerns regarding course completion, as less than 10% of the newborns treated for clinically-defined infections received the recommended antibiotics for 7 days or more. Injudicious use of antibiotics may lead to antibiotic resistance which is a critical public health concern in both resource-rich and resource-poor settings [62, 63]. Inappropriate provision or overuse of antibiotics also brings an economic burden on the health system and families through out-of-pocket expenditure [64]. Knowledge gaps among the doctors and patients’ expectations and lack of understanding of the importance of completing an antibiotic course by the family members may explain this inappropriate and irrational use of antibiotics for treating infections [65, 66].

In the EN-BIRTH study, verification of hospital inpatient case notes revealed that almost all (97%) newborns admitted in the hospital for clinically-defined infections received injectable antibiotics. However, very few (< 2%) had laboratory-confirmed evidence of any infection in Bangladesh and Tanzania. Importantly, in Nepal, there was a much higher rate of blood culture. Most likely this has happened as a result of the ongoing quality improvement initiatives in Nepal. Diagnostic tests are vital for managing newborns with infections [13, 67]. It is also an important aspect of antibiotic stewardship. Almost none of the newborns treated for clinically-defined infections in Azimpur BD, Kushtia BD, Temeke TZ and Muhimbili TZ had laboratory-confirmed evidence of any infection. Other supportive lab diagnoses such as a complete blood count (CBC) or WBC count were also not performed in Azimpur BD, Kushtia BD and Temeke TZ. This gap in diagnostic tests may be the result of inadequate provision of laboratory services in these resource-poor settings [68–70]. Ensuring the basic laboratory services with quality and standardisation in referral hospitals should be prioritised for improving the quality of care. It is important to explore and understand the enablers of such practices in Pokhara NP, and adapt learning for use in hospitals with similar settings.

### Strengths and limitations

Our study has strengths, notably the large sample size and multi-country sites with standardised tools and training and a custom-built android tablet-based application that was designed specifically for this study [71]. Data abstraction from inpatient case notes was conducted by trained study nurses, supervised by study-physicians. Exit surveys with women were conducted by trained data collectors. These measures helped to ensure multi-site consistency and data quality through real-time monitoring.

It is also important to acknowledge limitations. Observations of the clinical practices in the selected hospitals and especially timed observations of antibiotic administered for neonatal infections were not feasible, and hence we used inpatient case notes as the gold standard to assess the validity of women’s report through the exit survey. Whilst this is the most commonly used gold standard in many validation studies, It is widely recognised that case note documentation has gaps, even in well-resourced settings [72]. Validity assessment may be affected by the potential inaccuracy of case note documentation however, we note that case notes are more likely to omit than have false record of giving treatment, so if anything our findings are conservative, and the gap between “truth” and reported coverage may be even higher. Within the quality gap analyses gaps may be related to documentation as well as gaps in quality of care. The survey and our analyses were limited to cases admitted for infection; therefore, we could not compare the true negatives for women’s report.

## Conclusion

Survey report consistently underestimated the coverage of injectable antibiotics for treating newborns with infection, and had low sensitivity for both the numerator and denominator, hence we recommend this indicator not be added to population-based surveys. However, the high sensitivity of a “contact” point indicator of admission to a neonatal unit, at least amongst those admitted, holds promise for tracking coverage of small and sick newborn care. More investment and research on hospital inpatient records for newborns is crucial to enable linked data on content and quality of care. We particularly recommend improving the design of inpatient registers and case notes to address the identified gaps in measurements of quality of care. Strengthening capacities to do blood cultures and lumbar punctures is important in the short term, and in the longer term novel bedside diagnostics for bacterial and viral neonatal infections could be transformative and also to improve antibiotic stewardship and address AMR.

## Supplementary Information

**Additional file 1.** EN-BIRTH study data collection dates by site and time elapsed between birth and exit survey.

**Additional file 2.** Ethical approval of local institutional review boards, EN-BIRTH study.

**Additional file 3. **STROBE Statement—Checklist of items that should be included in reports of observational studies.

**Additional file 4. **EN-BIRTH study background characteristics of the mothers of the newborns, exit interview survey (*n* = 910 mothers).

**Additional file 5. **Neonatal infection individual-level validation two-way tables, EN-BIRTH study, Neonatal infection dataset (*n* = 1015, stratified by site).

**Additional file 6. **Neonatal infection indicator individual-level validation results, EN-BIRTH study, Neonatal infection dataset (*n* = 1015, stratified by site).

## Data Availability

The datasets generated during and/or analysed during the current study are available on LSHTM Data Compass repository, https://datacompass.lshtm.ac.uk/955/.

## Abbreviations

AMR: Antimicrobial resistance
CBC: Complete blood count
BD: Bangladesh
CIFF: Children’s Investment Fund Foundation
CSF: Cerebrospinal Fluid
DHS: The Demographic and Health Surveys Program
ENAP: *Every Newborn* Action Plan now branded as *Every Newborn*
EN-BIRTH: *Every Newborn*-Birth Indicators Research Tracking in Hospitals study
g: grammes
HMIS: Health Management Information Systems
icddr,b: International Centre for Diarrheal Disease Research, Bangladesh
IHI: Ifakara Health Institute, Tanzania
LMIC: Low- and middle-income countries
LP: lumbar puncture
LSHTM: London School of Hygiene & Tropical Medicine
MCHTI: Maternal & Child Health Training Institute, Azimpur, Bangladesh
MICS: Multiple Indicator Cluster Surveys
MUHAS: Muhimbili University of Health and Allied Sciences, Tanzania
NP: Nepal
PRISM: Performance of Routine Information System Management
SDG: Sustainable Development Goals
SQL: Structured Query Language
TZ: Tanzania
WBC: White Blood Cell
WHO: World Health Organization
