# Supplementary material for: Antibiotic use for inpatient newborn care with suspected infection: EN-BIRTH multi-country validation study
Source: BMC Pregnancy Childbirth. 2021 Mar 26;21(Suppl 1):229. doi: 10.1186/s12884-020-03424-7 (PMC7995687; doi:10.1186/s12884-020-03424-7)
Supplement: Supplementary file 4 — Additional file 4. EN-BIRTH study background characteristics of the mothers of the newborns, exit interview survey (n = 910 mothers). [file 12884_2020_3424_MOESM4_ESM.pdf]

Antibiotic use for inpatient newborn care with suspected infection: EN-BIRTH multi-country validation study

Additional File 4: EN-BIRTH study background characteristics of the mothers of the newborns, exit interview survey (n=910 mothers)

	Bangladesh		Nepal	Tanzania	
	**Azimpur Tertiary**	**Kushtia District**	**Pokhara Regional**	**Temeke Regional**	**Muhimbili National**
	**N=104**	**N=302**	**N=318**	**N=151**	**N=35**
	**n (%)**	**n (%)**	**n (%)**	**n (%)**	**n (%)**
**Age**					
<18 years	3(2.9)	6(2)	13(4.1)	2(1.3)	1(2.9)
18-19 years	23(22.1)	44(14.6)	36(11.3)	12(7.9)	3(8.6)
20-24 years	39(37.5)	118(39.1)	117(36.8)	46(30.5)	9(25.7)
25-29 years	27(26)	76(25.2)	94(29.6)	42(27.8)	12(34.3)
30-34 years	9(8.7)	40(13.2)	46(14.5)	31(20.5)	5(14.3)
35+ years	3(2.9)	18(6)	12(3.8)	18(11.9)	5(14.3)
**Education**					
No Education	7(6.7)	0(0)	11(3.5)	9(6)	4(11.4)
Primary incomplete	2(1.9)	20(6.6)	10(3.1)	1(0.7)	3(8.6)
Primary complete	15(14.4)	43(14.2)	17(5.3)	1(0.7)	0(0)
Secondary incomplete	30(28.8)	143(47.4)	70(22)	93(61.6)	12(34.3)
Secondary complete	48(46.2)	87(28.8)	194(61)	46(30.5)	16(45.7)
Don't know	2(1.9)	9(3)	16(5)	1(0.7)	0(0)
**Religion**					
Muslim	100(96.2)	293(97)	3(0.9)	-	-
Hindu	4(3.8)	8(2.6)	299(94)	-	-
Buddhist	0(0)	1(0.3)	11(3.5)	-	-
Christian	0(0)	0(0)	5(1.6)	-	-
**Wealth Quintile**					
Lowest	1(1)	127(42.1)	66(20.8)	37(24.5)	7(20)
Second	4(3.8)	127(42.1)	52(16.4)	57(37.7)	6(17.1)
Middle	31(29.8)	32(10.6)	73(23)	24(15.9)	7(20)
Fourth	34(32.7)	11(3.6)	78(24.5)	22(14.6)	8(22.9)
Highest	33(31.7)	5(1.7)	45(14.2)	6(4)	7(20)
Couldn't define	1(1)	0(0)	4(1.3)	5(3.3)	0(0)
