# Supplementary material for: Antibiotic use for inpatient newborn care with suspected infection: EN-BIRTH multi-country validation study
Source: BMC Pregnancy Childbirth. 2021 Mar 26;21(Suppl 1):229. doi: 10.1186/s12884-020-03424-7 (PMC7995687; doi:10.1186/s12884-020-03424-7)
Supplement: Supplementary file 5 — Additional file 5. Neonatal infection individual-level validation two-way tables, EN-BIRTH study, Neonatal infection dataset (n = 1015, stratified by site). [file 12884_2020_3424_MOESM5_ESM.pdf]

***Every Newborn* BIRTH multi-country validation study: informing measurement of coverage and quality of maternal and newborn care**

Antibiotic use for inpatient newborn care with suspected infection: EN-BIRTH multi-country validation study

Additional File 5: Neonatal infection individual-level validation two-way tables, EN-BIRTH study, Neonatal infection dataset (n=1015, stratified by site)

				Bangladesh		Nepal	Tanzania	
				Azimpur Tertiary	Kushtia District	Pokhara Tertiary	Temeke District	Muhimbili Tertiary
**5.1 Neonatal Infection Antibiotics - Injection**		**Gold Standard**	**2x2 table classify**					
	Survey reported	Antibiotic treatment	True Positive	84	163	138	127	31
		Antibiotic treatment	False Positive	0	5	9	4	0
		No Antibiotics	True Negative	1	3	10	1	0
		No Antibiotics	False Negative	6	16	79	1	0
	Survey "Don't know"	Antibiotic treatment	Positive	10	103	72	8	4
		No Antibiotics	Negative	0	3	5	1	0
**5.2 Neonatal Infection Antibiotics - name**		**Gold Standard**	**2x2 table classify**					
	Survey reported	Antibiotic treatment	True Positive	5	74	10	29	5
		Antibiotic treatment	False Positive	0	1	0	1	0
		No Antibiotics	True Negative	1	7	19	4	0
		No Antibiotics	False Negative	85	105	207	99	26
	Survey "Don't know"	Antibiotic treatment	Positive	10	103	72	8	4
		No Antibiotics	Negative	0	3	5	1	0
