# Supplementary material for: Antibiotic use for inpatient newborn care with suspected infection: EN-BIRTH multi-country validation study
Source: BMC Pregnancy Childbirth. 2021 Mar 26;21(Suppl 1):229. doi: 10.1186/s12884-020-03424-7 (PMC7995687; doi:10.1186/s12884-020-03424-7)
Supplement: Supplementary file 6 — Additional file 6. Neonatal infection indicator individual-level validation results, EN-BIRTH study, Neonatal infection dataset (n = 1015, stratified by site). [file 12884_2020_3424_MOESM6_ESM.pdf]

Every Newborn BIRTH multi-country validation study: informing measurement of coverage and quality of maternal and newborn care

Antibiotic use for inpatient newborn care with suspected infection: EN-BIRTH multi-country validation study

Additional File 6: Neonatal infection indicator individual-level validation results, EN-BIRTH study, Neonatal infection dataset (n=1015), stratified by site

	Bangladesh				Nepal		Tanzania				All sites						
	Azimpur Tertiary		Kushtia District		Pokhara Regional		Temeke Regional		Muhimbili National		Pooled Random Effects		Q	df	p-val.	i²	τ²
**5.1 Neonatal Infection - Antibiotic/Injection - Survey reported**																	
Observer coverage %	99.0	(93.4,99.9)	96.3	(93.4,97.9)	92.6	(89.3,95.0)	95.6	(91.8,97.7)	100.0	(92.5,100.0)	96.7	(94.0,98.6)	13.3	4	0.010	70.0%	0.013
Survey reported coverage %	82.5	(73.9,88.7)	58.1	(52.5,63.6)	46.8	(41.4,52.4)	91.1	(85.2,94.8)	88.6	(72.9,95.7)	74.7	(55.3,90.1)	137.5	4	<0.001	97.1%	0.204
"Don't know" responses %	9.7	(5.3,17.2)	35.2	(30.0,40.8)	25.0	(20.5,30.1)	6.8	(3.7,12.3)	11.4	(4.3,27.1)	16.9	(7.4,29.2)	69.3	4	<0.001	94.2%	0.100
**Current survey - Count "don't know" as "no"**																	
Sensitivity % (95% CI)	†	†	57.8	(51.8,63.6)	47.8	(41.9,53.7)	†	†	†	†	75.9	(55.6,91.6)	144.0	4	<0.001	97.2%	0.228
Specificity % (95% CI)	‡	‡	‡	‡	‡	‡	‡	‡	‡	‡	‡	‡	‡	‡	‡	‡	‡
Percent agreement (TP+TN)/n (95% CI)	84.2	(75.6,90.7)	57.7	(51.8,63.4)	48.9	(43.2,54.6)	90.8	(84.9,95.0)	88.6	(73.3,96.8)	75.3	(56.4,90.2)	128.8	4	<0.001	96.9%	0.194
Positive Predictive Value % (95% CI)	†	†	97.0	(93.2,99.0)	93.9	(88.7,97.2)	†	†	†	†							
Negative Predictive Value % (95% CI)	†	†	4.8	(1.8,10.2)	9.0	(5.1,14.5)	†	†	†	†							
**Consider only "yes" and "no" (exclude don't know)**																	
Sensitivity (95% CI)	†	†	†	†	63.6	(56.8,70.0)	†	†	†	†	92.4	(76.7,99.9)	119.4	4	<0.001	96.7%	0.238
Specificity (95% CI)	‡	‡	‡	‡	‡	‡	‡	‡	‡	‡	‡	‡	‡	‡	‡	‡	‡
Percent agreement (TP+TN)/n (95% CI)	93.4	(86.0,97.0)	88.8	(83.4,92.6)	62.7	(56.3,68.7)	96.2	(91.2,98.4)	100.0	(0.0,0.0)	90.6	(75.7,99.1)	104.0	4	<0.001	96.2%	0.197
Positive Predictive Value (95% CI)	†	†	†	†	93.9	(88.7,97.2)	†	†	†	†							
Negative Predictive Value (95% CI)	†	†	†	†	11.2	(5.5,19.7)	†	†	†	†							
**5.2 Neonatal Infection - Antibiotic name - Survey reported**																	
Observer coverage %	99.0	(93.4,99.9)	96.3	(93.4,97.9)	92.6	(89.3,95.0)	95.6	(91.8,97.7)	100.0	(92.5,100.0)	96.7	(94.0,98.6)	13.3	4	0.010	70.0%	0.013
Survey reported coverage %	4.9	(2.0,11.2)	25.2	(20.6,30.5)	3.2	(1.7,5.8)	21.2	(15.3,28.7)	14.3	(6.0,30.4)	12.3	(3.5,25.1)	89.1	4	<0.001	95.5%	0.130
"Don't know" responses %	9.7	(5.3,17.2)	35.2	(30.0,40.8)	25.0	(20.5,30.1)	6.8	(3.7,12.3)	11.4	(4.3,27.1)	16.9	(7.4,29.2)	69.3	4	<0.001	94.2%	0.100
**Current survey - Count "don't know" as "no"**																	
Sensitivity % (95% CI)	†	†	26.2	(21.2,31.8)	3.5	(1.7,6.3)	†	†	†	†	12.7	(3.7,25.6)	83.5	4	<0.001	95.2%	0.129
Specificity % (95% CI)	‡	‡	‡	‡	‡	‡	‡	‡	‡	‡	‡	‡	‡	‡	‡	‡	‡
Percent agreement (TP+TN)/n (95% CI)	5.9	(2.2,12.5)	28.7	(23.6,34.2)	10.9	(7.6,14.8)	23.9	(17.2,31.8)	14.3	(4.8,30.3)	16.1	(8.0,26.2)	48.7	4	<0.001	91.8%	0.070
Positive Predictive Value % (95% CI)	†	†	98.7	(92.8,100.0)	100.0	(69.2,100.0)	†	†	†	†							

Negative Predictive Value % (95% CI)	†	†	4.6	(2.2,8.3)	7.9	(5.1,11.6)	†	†	†	†							
**Consider only "yes" and "no" (exclude don't know)**																	
Sensitivity (95% CI)	†	†	†	†	4.6	(2.2,8.3)	†	†	†	†	16.2	(4.0,33.9)	104.1	4	<0.001	96.2%	0.206
Specificity (95% CI)	‡	‡	‡	‡	‡	‡	‡	‡	‡	‡	‡	‡	‡	‡	‡	‡	‡
Percent agreement (TP+TN)/n (95% CI)	6.6	(2.5,13.8)	43.3	(36.1,50.7)	12.3	(8.4,17.2)	24.8	(17.7,33.0)	16.1	(5.5,33.7)	19.5	(7.8,34.6)	73.9	4	<0.001	94.6%	0.138
Positive Predictive Value (95% CI)	†	†	†	†	100.0	(69.2,100.0)	†	†	†	†							
Negative Predictive Value (95% CI)	†	†	†	†	8.4	(5.1,12.8)	†	†	†	†							

N/A= data element not captured by routine register
‡= specificity not reported as all true negatives not captured
TP= true positive. TN= true negative
